# Identification and Characterization of Antigenic Properties of *Schistosoma japonicum* Heat Shock Protein 90α Derived Peptides

**DOI:** 10.3390/pathogens11111238

**Published:** 2022-10-26

**Authors:** Chunxiang Shen, Xinyi Zhu, Xuejun Xu, Hao Chang, Yangyue Ni, Chen Li, Kaiyue He, Lin Chen, Lu Chen, Min Hou, Minjun Ji, Zhipeng Xu

**Affiliations:** 1 Department of Pathogen Biology, Jiangsu Province Key Laboratory of Modern Pathogen Biology, Nanjing Medical University, Nanjing 211166, China; 2 State Key Laboratory of Reproductive Medicine, Nanjing 211166, China; 3 NHC Key Laboratory of Antibody Technique, Nanjing Medical University, Nanjing 211166, China

**Keywords:** heat shock protein, peptide, *Schistosoma japonicum*, follicular helper T cells

## Abstract

It is known that schistosome-derived antigens induce innate and adaptive immune responses that are essential for the formation of hepatic immunopathology. Here, we screened and synthesized four peptides derived from *Schistosoma japonicum* (*S. japonicum*) heat shock protein 90α (Sjp90α-1, -2, -3, and -4), which is widely expressed in adults and eggs of the genus *S. japonicum* and induces remarkable immune reactions. To define the antigenicity of these peptides, we stimulated splenocytes with peptides, and the results showed that only the Sjp90α-1 peptide could predominately induce the activation of dendritic cells (DCs) and macrophages as well as alter the proportion of follicular helper T (Tfh) cells. Next, CD4^+^ T cells were purified and cocultured with mouse bone-marrow-derived DCs (BMDCs) with or without Sjp90α-1 peptide stimulation *in vitro*, and the results showed that Sjp90α-1-stimulated BMDCs can significantly induce CD4^+^ T-cell differentiation into Tfh cells, while the direct stimulation of CD4^+^ T cells with Sjp90α-1 did not induce Tfh cells, indicating that the Sjp90α-1 peptide promotes Tfh cell differentiation depending on the presence of DCs. Furthermore, we selected and prepared an Sjp90α-1-peptide-based antibody and illustrated that it has excellent reactivity with the immunizing peptide and detects a single band of 29 kDa corresponding to the Sjp90α protein. The immunolocalization results showed that the protein recognized by this Sjp90α-1-peptide-based antibody is present in the mature eggs and the tegument of adults, implying that the parasite-derived peptide has a potential interaction with the host immune system. Finally, we evaluated antipeptide IgG antibodies and revealed a significantly higher level of anti-Sjp90α-1 peptide IgG antibodies in mice 3 weeks after *S. japonicum* infection. In conclusion, we illustrate that these synthetic peptides warrant further investigation by evaluating their antigen-specific immune response and their ability to efficiently induce Tfh cells. Moreover, they may constitute a potentially helpful method for the laboratory diagnosis of schistosomiasis japonica.

## 1. Introduction

Schistosomiasis, caused by the blood fluke schistosome, is one of the most prevalent, insidious, and serious zoonotic parasitic diseases in the world, which in China is mainly transmitted by *Schistosoma japonicum* (*S. japonicum*) [1]. It affects more than 140 million individuals and causes approximately 200,000 annual deaths worldwide [2]. Traditional parasitological detection methods such as the Kato–Katz (KK) coproparasitological test, exhibit low sensitivity in areas with reduced prevalence/disease intensity [3,4], and serologic detection primarily using a crude extracted antigen presents wide-scale cross-reactivity [5]. The identification of highly specific schistosome antigens with a high level of sensitivity is thus a prerequisite for developing serological diagnostics. During *S. japonicum* infection, eggs excrete a range of molecules from their excretory pores. These are jointly referred to as excretory–secretory products (ESP), including heat shock proteins (HSPs), which have been shown to induce strong immunomodulatory effects, including immunostimulatory reactivity [6,7]. *S. japonicum* heat shock protein 90α (Sjp90α, the inducible cytosolic isoform of Hsp90), one of the most abundant proteins from *S. japonicum* [8], is present at high concentrations in the ESP of adult *S. japonicum,* according to a proteome analysis [9]. Our previous work showed that Sjp90α has potential antigenicity and immunogenicity [10]. However, the exact fragments of Sjp90α protein that exert potential immunogenicity and diagnosis are still unknown. 

Peptides play vital roles in driving the adaptive immune response as the immunogenic epitopes [11], which are mediated primarily by their interaction with major histocompatibility complexes (T-cell epitopes) and antibodies (B-cell epitopes) [12,13]. Moreover, peptides have been employed in a variety of assays to detect parasitic infections; for instance, SjSAP4-derived peptides [14], Gp63 peptides [15], and HLA–peptide complexes [16] were identified for the serological diagnosis of schistosomiasis or vaccine formulation as malaria prophylaxis. However, studies on the potential immunological effects of Sjp90α-derived peptides have not been reported. 

Antigen-presenting cells such as B cells, macrophages, and dendritic cells are activated by schistosome worm/egg antigens during the early phase of the innate activated immune response and play critical roles in the adaptive immune response during *S. japonicum* infection [17]. It is known that T lymphocytes, especially CD4^+^ T cells, are also essential for immune responses against *Schistosoma* species [18,19]. CD4^+^ T-cell subsets have been classified into several distinct T-helper (Th) phenotypes, including Th1, Th2, Th17, T follicular helper cells (Tfh), and regulatory T cells (Tregs) [20]. Among these cell subsets, Tfh cells, which can be identified by the expression of various molecules, such as the surface receptors CXCR5 and programmed death 1 (PD-1), are mainly located in the periphery of B-cell follicles and are critical for the activation of B cells, antibody production, and germinal center (GC) formation [21]. Therefore, it would be interesting to test whether Sjp90α-derived peptides could induce the immune system to produce an immunological effect. 

In the present study, we predicted four peptides screened by antigenicity and B-cell epitope prediction and evaluated the potential immunological effects of the peptides on immune cells, including macrophages, dendritic cells (DCs), and T-cell subsets. Additionally, immunolocalization studies of Sjp90α were undertaken in the eggs and worms based on an Sjp90α-1-peptide-based antibody. Furthermore, the peptide-based ELISA was further evaluated for the serodiagnosis of *S. japonicum* using the Sjp90α-1 peptide as the antigenic base. Altogether, this study provides crucial molecular information about Sjp90α-derived peptides and contributes to a promising alternative approach for diagnosing schistosomiasis.

## 2. Results

### 2.1. Design and Preparation of Sjp90α-Derived Peptides

**Figure 1:**
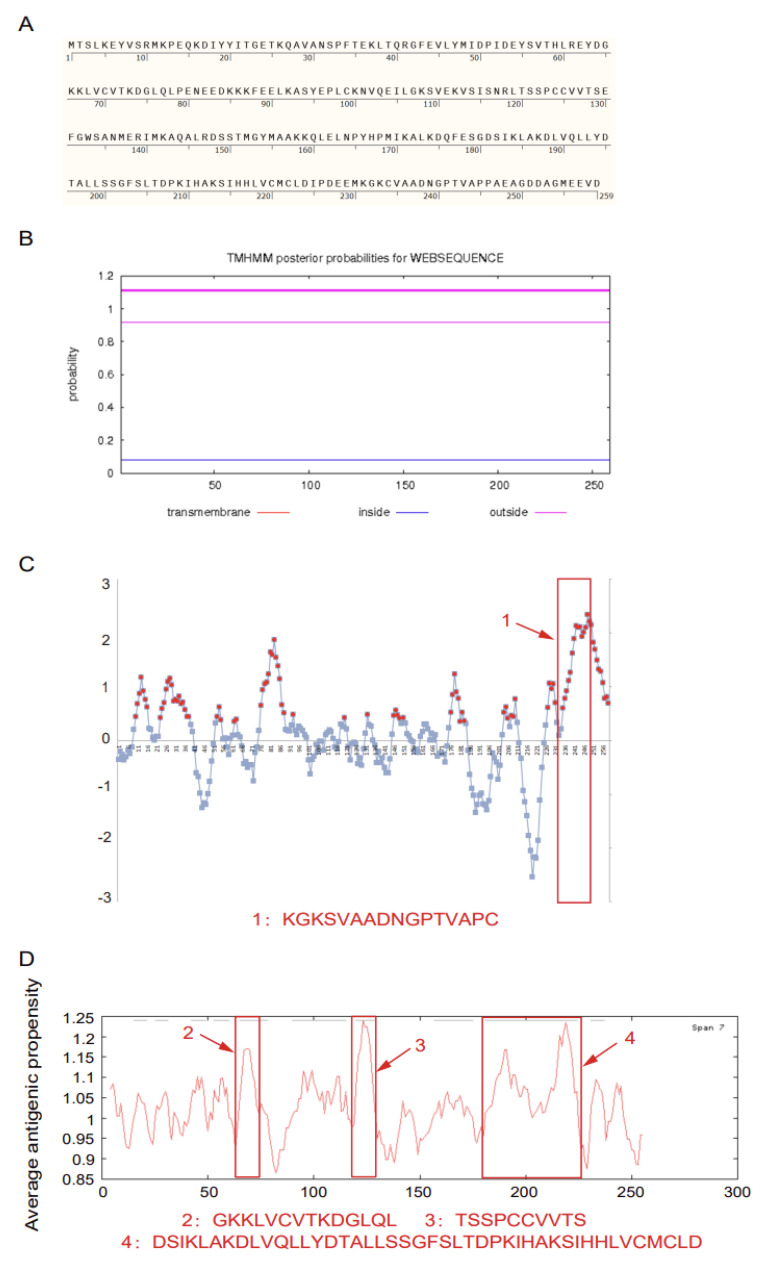
Evaluation of Sjp90α and prediction of Sjp90α-derived peptides. (**A**) The protein sequence of Sjp90α is shown in SnapGene Viewer (https://www.snapgene.com/snapgene-viewer/, accessed on 21 October 2022). (**B**) The amino acid sequences of Sjp90α were analyzed to predict transmembrane structures using TMHMM Server v.2.0. No transmembrane domains were predicted in this protein structure (http://www.cbs.dtu.dk/services/TMHMM/, accessed on 21 October 2022). (**C**) B-cell epitope prediction of the most antigenic protein. The x-axis and y-axis represent the sequence position and antigenic propensity, respectively. The threshold value is 0. The regions above the threshold are antigenic, shown as red dots, while the blue color reflects the peptide regions that could not satisfy the threshold margin. (**D**) Average antigenic propensity plot result for the Sjp90α sequence. Residues with propensity index values above 1.0 are potentially antigenic (the reported accuracy of this method is about 75%).

The sequence of Sjp90α comprises an open reading frame (ORF) of 780 bp encoding 259 amino acids. The full-length amino acid sequence is shown using SnapGene Viewer (Figure 1A). The optimal peptides were screened by considering the sequence length, hydrophilicity/ hydrophobicity, sequence specificity, and epitope properties. First, transmembrane-derived peptides have a hydrophobic nature, which is hard to handle and unsuitable for synthesis [22]. Moreover, the transmembrane domain may further influence their antigenicity [23]. Therefore, we predicted the protein transmembrane region by TMHMM Server (Figure 1B) and found that Sjp90α does not contain transmembrane structures, indicating that the peptides could be suitable for synthesis. We used a DTU service for B-cell epitope prediction and obtained the peptide (231-245aa) named Sjp90α-1 (Figure 1C). The peptide designs were also made by predicting antigenicity using Kolaskar and Tongaonkar [24], and three peptides were selected, named Sjp90α-2, Sjp90α-3, and Sjp90α-4 (Figure 1D). In addition, we tested the amino acid sequence similarity of the identified peptides in *Schistosoma* species using the BLASTP program (http://www.ncbi.nlm.nih.gov/Blast.cgi, accessed on 21 October 2022), and the analysis revealed a high level of homology (>90%) between Sjp90α-2, -3, -4, and the protein of *Schistosoma mansoni*, while Sjp90α-1 contains a unique amino acid sequence that is specific to *Schistosoma japonicum*.

### 2.2. Effects of Sjp90α-Derived Peptides on Splenocytes 

**Figure 2:**
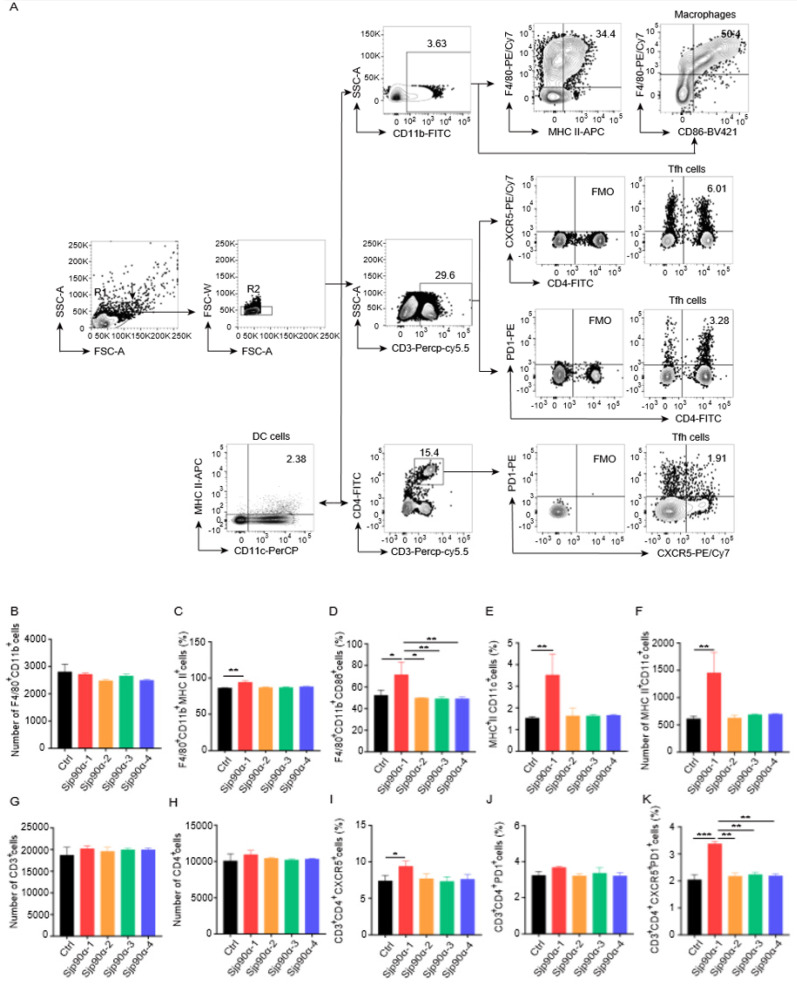
Different effects of Sjp90α-derived peptides on immune cells. Mouse splenocytes were treated with Sjp90α-derived peptides (40 µg/mL) (n = 4–5 wells, in quadruplicate or quintuplicate) or PBS for 24 h and analyzed by flow cytometry. The gating strategy (**A**) and statistical chart (**B**–**K**) showed the activation of macrophages (F4/80^+^CD11b^+^MHC-II^+^ and F4/80^+^CD11b^+^CD86^+^) and DCs (MHC-II^+^CD11c^+^) as well as the frequencies of Tfh cells (CD3^+^CD4^+^CXCR5^+^; CD3^+^CD4^+^PD1^+^; and CD3^+^CD4^+^CXCR5^+^PD1^+^). Data are presented as means ± SEM. * *p* < 0.05; ** *p* < 0.01; *** *p* < 0.001. The data are from one representative experiment out of two experiments with similar results.

To better understand the roles of different cell types in the immune responses to Sjp90α-derived peptides, we initially tested cell viability at different peptide concentrations and found that 40 µg/mL was the optimal peptide concentration (data not shown). Then we stimulated mouse splenocytes in vitro with each Sjp90α-derived peptide. FACS analysis indicated that only the Sjp90α-1, but not the Sjp90α-2, -3, or -4 peptides, significantly increased the activation of macrophages (F4/80^+^CD11b^+^MHC-II^+^ and F4/80^+^CD11b^+^CD86^+^) (Figure 2A–D) and dendritic cells (MHC-II^+^CD11c^+^) (Figure 2E,F). Nevertheless, all Sjp90α-derived peptides had no effect on NK cells and B cells (Supplementary Figure S1A–D).

T-cell subsets, including Th1, Th2, Th17, T follicular helper (Tfh), and T regulatory (Treg) cells, were evaluated after the stimulation of splenocytes with Sjp90α-derived peptides, and we fortuitously found that Tfh cells were enhanced after stimulation with the Sjp90α-1 peptide (Figure 2G–K). However, there was no significant change in the other T-cell subsets (Supplementary Figure S1E–H). Altogether, these data suggest that Sjp90α-1 peptide might be involved in the antigen-specific immune response.

### 2.3. Sjp90α-1 Peptide-Activated BMDCs Induce CD4^+^ T-Cell Differentiation into Tfh Cells

**Figure 3:**
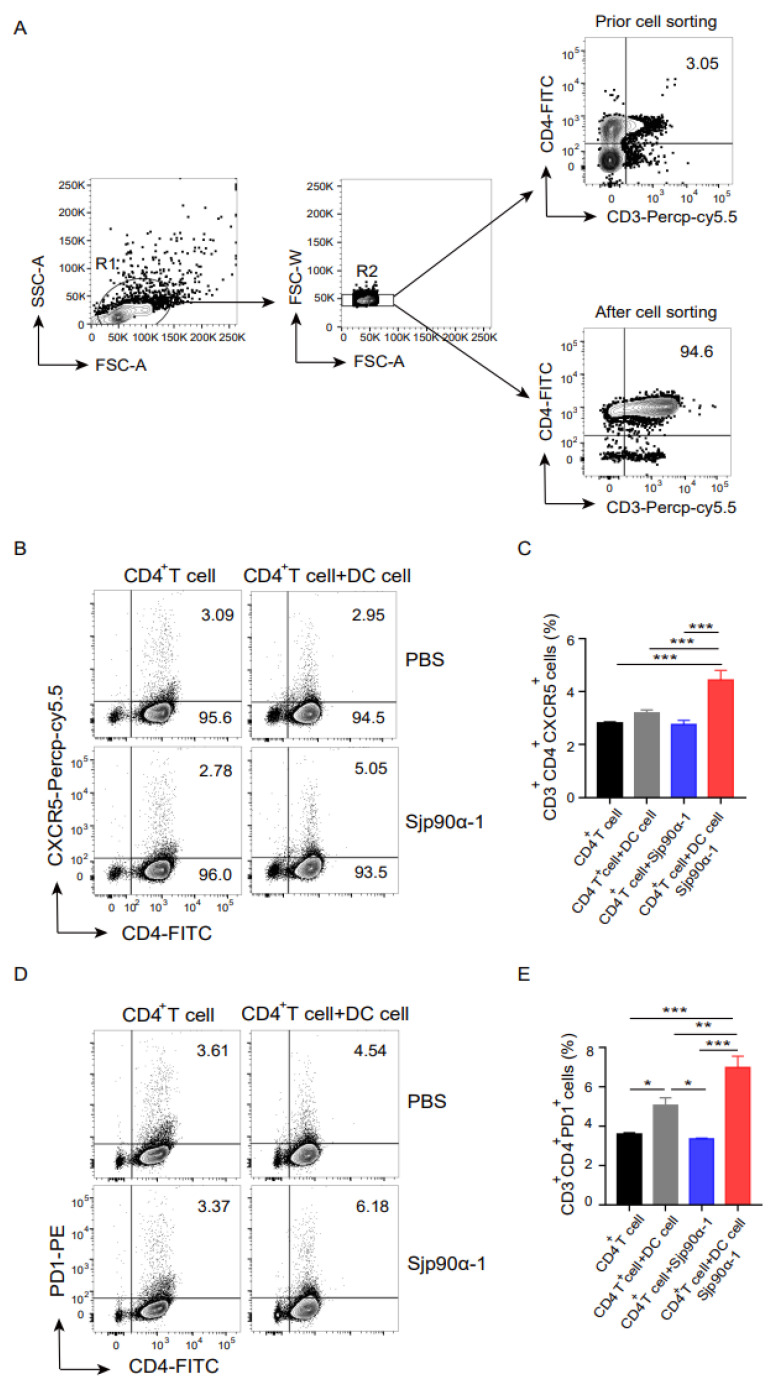
Sjp90α-1 regulates CD4^+^ T-cell differentiation through the activation of BMDCs. (**A**) Gating strategy for identifying the purified CD3^+^ CD4^+^ T cells. (**B**–**E**) Purified CD4^+^ T cells were cocultured with BMDCs with or without Sjp90α-1 peptide (40 µg/mL) stimulation and compared with purified CD4^+^ T cells. The proportion of Tfh cells was detected by flow cytometry after 24 h of coculture. Data are presented as means ± SEM. * *p* < 0.05; ** *p* < 0.01; *** *p* < 0.001. The data are from one representative experiment out of two experiments with similar results.

To investigate whether the induction of CD4^+^ T-cell differentiation into Tfh cells with Sjp90α-1 peptide was dependent on antigen-presenting cells, we purified CD4^+^ T cells and cocultured them with bone-marrow-derived dendritic cells (BMDCs) with or without Sjp90α-1 peptide stimulation for 24 h (Figure 3A), and the results showed that Sjp90α-1-activated BMDCs were essential to induce CD4^+^ T-cell differentiation into Tfh cells (Figure 3B–E), which are increased in the acute schistosomiasis patients and an *S. japonicum*-infected mouse model [25,26,27,28]. However, the direct stimulation of CD4^+^ T cells with Sjp90α-1 *in vitro* did not induce their differentiation into Tfh cells. Taken together, these data indicate that the Sjp90α-1 peptide promotes CD4^+^ T-cell differentiation into Tfh cells, dependent on BMDCs. 

### 2.4. Preparation and Evaluation of Sjp90α-1-Peptide-based Antibody 

**Figure 4:**
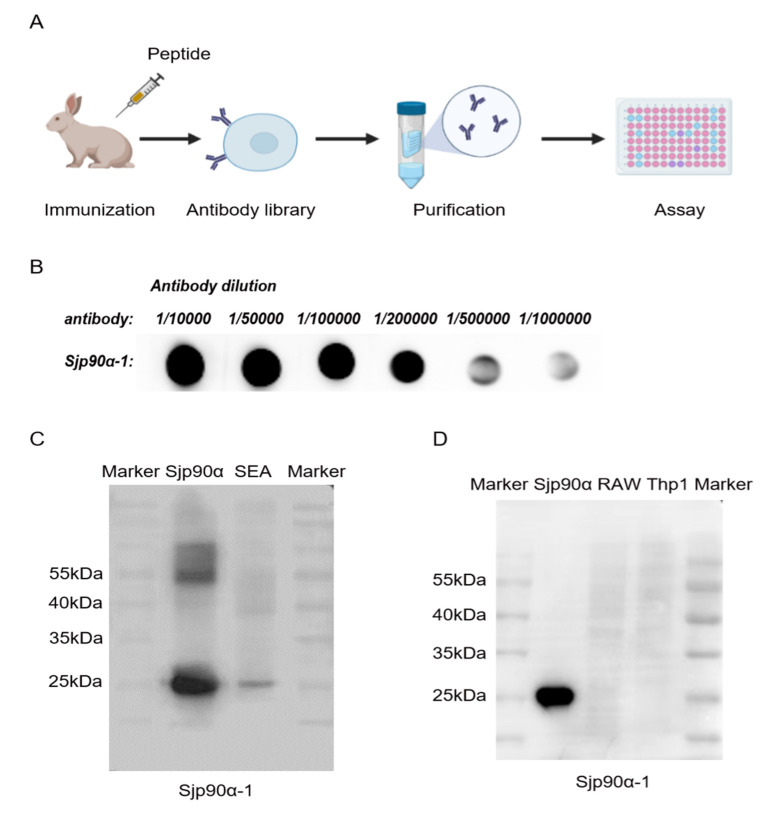
Production of Sjp90α-1-peptide-based antibody and detection of the antibody sensitivity. (**A**) Pattern diagram of peptide antibody preparation. Two Japanese white rabbits were immunized with the Sjp90α-1 peptide, and the serum was collected and screened in the antibody library. Finally, the antibody was affinity-purified. The obtained polyclonal antibody was verified by an immunological assay. (**B**) Sjp90α-1 peptide (100 ng) was loaded onto a nitrocellulose (NC) membrane. The antibody was diluted into different proportions and probed on NC membranes for incubation. (**C**) The specificity of the Sjp90α-1-peptide-based antibody was determined by Western blot analysis using Sjp90α and SEA from *S. japonicum*. (**D**) The cross-reactivity of the antibody was detected by Western blot analysis in mouse mononuclear macrophages (RAW264.7 cells) and human mononuclear cells (Thp1).

Given that the Sjp90α-1 peptide (KGKCSVAADNGPTVAP-C) is potentially highly immunogenic, we next produced antibodies against this peptide. Antibodies against the Sjp90α-1 were raised in the rabbit within 10 weeks (Figure 4A), and the high antisera titer after immunization was tested by the antiserum DB (dot blot hybridization) (Figure 4B). The immunogenicity of the Sjp90α-1-peptide-based antibody was assessed by Western blot, and the results showed a band at 29 kDa (Figure 4C), indicating the affinity of the antibody against the Sjp90α-1 peptide towards the whole Sjp90α protein. Furthermore, no cross-reactivity of the antibody was observed against Sjp90α-1 when using mouse mononuclear macrophages (RAW264.7 cells) and human mononuclear cells (Thp1) as controls (Figure 4D). Altogether, these results reveal the specific immune recognition of the Sjp90α-1-peptide-based antibody.

### 2.5. Distribution of Sjp90α in S. japonicum Eggs and Adults

**Figure 5:**
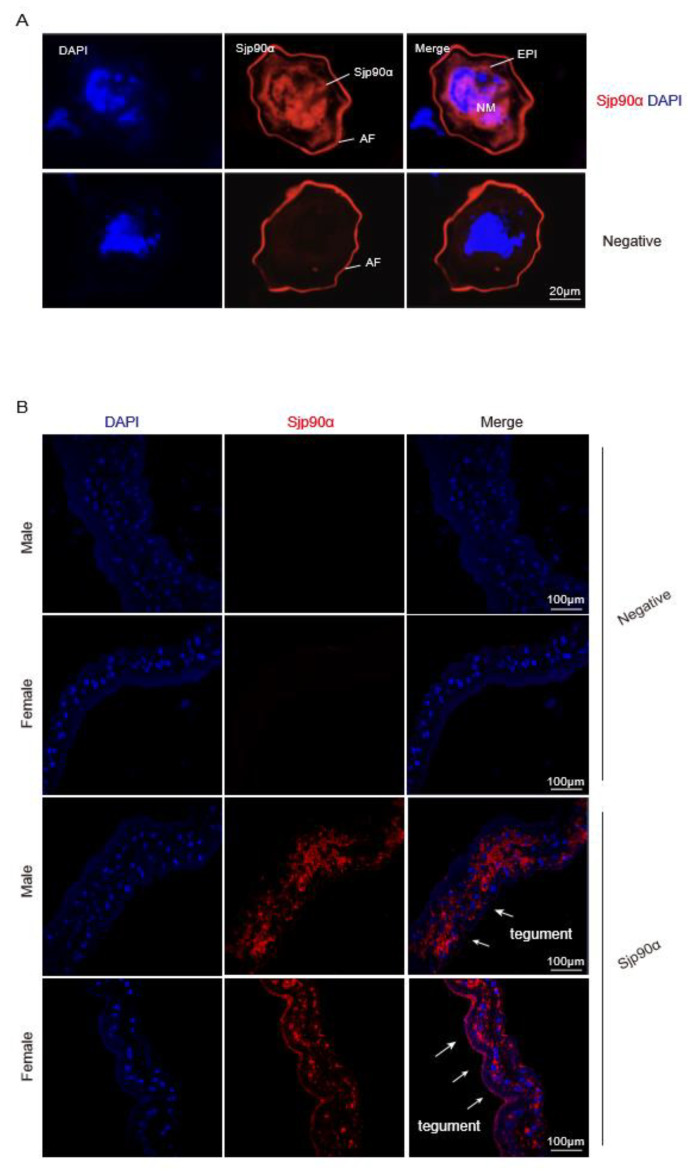
Immunolocalization of Sjp90α in eggs trapped in infected mouse liver probed with Sjp90α-1-peptide-based antibody. Sections of eggs (**A**) and worms (**B**) from mice infected with *S. japonicum* were labeled with Sjp90α-1-peptide-based antibody coupled with Alexa-Fluor 647 goat antirabbit IgG (red). DAPI was used to stain for nuclei, and the samples were analyzed using a Zeiss 780 NLO laser. Negative control sections of the egg and worm were incubated with naïve control rabbit serum. NM—neural mass; EPI—epidermal cells; AF—autofluorescence. Scale bars: 20 µm. White arrows indicate the Sjp90α expressed in the tegument of worms. Scale bars: 100 µm.

The immunolocalization of eggs trapped in the infected mouse livers was observed using immunofluorescence based on the Sjp90α-1-peptide-based antibody. The results showed that Sjp90α mainly localized to neural mass (NM) cells (a single large cell with numerous peripheral nuclei) and the epidermis (EPI) cells of the intraovular miracidium within mature eggs (Figure 5A). In addition, we also determined the distribution of Sjp90α in the worms using an Sjp90α-1-peptide-based antibody, and the results showed that Sjp90α is also present outside the underlying musculature of worms, especially in the tegumental membranes of females (Figure 5B), indicating that the parasitic expression of tegumental Sjp90α might serve as the primary interface between the parasite and the host and mediate the communication of the worm with its host, which could be involved in the activation of the host immune response [29].

### 2.6. Dynamics of Sjp90α-1-Peptide-Based Antibody in Host Serum Determined by ELISA

**Figure 6:**
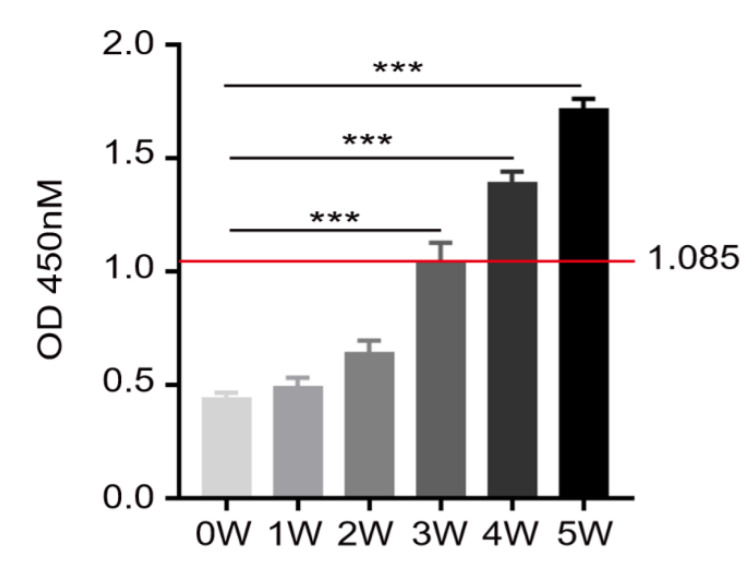
OD values of the IgG antibody in the ELISA test for infected mice. C57BL/6J mice were percutaneously infected with cercariae (12 ± 1 cercariae per mouse). Sera from the infected mice were collected on weeks 0, 1, 2, 3, 4, and 5 after infection. An Sjp90α-1-peptide-coated ELISA plate was used to detect the expression of the antibody in the serum of infected mice at different periods. The cutoff value for ELISA absorbance (1.085) is indicated with a horizontal line (mean + 2SD). Data are presented as means ± SEM. Statistical significance was calculated using a repeated-measures ANOVA test. *** *p* < 0.001. The data are from one representative experiment out of three experiments with similar results. The experiments each had n = 8 mice per group.

To evaluate the roles of the Sjp90α-1 peptide in the diagnosis of schistosomiasis, we coated plates with the Sjp90α-1 peptide and detected an antipeptide IgG antibody in serum from *S. japonicum*-infected mice by ELISA (Figure 6). The results showed a significant increase in the levels of the anti-Sjp90α-1 peptide IgG antibody three weeks after *S. japonicum* infection, suggesting its potential value for the early diagnosis of schistosomiasis.

## 3. Discussion

Animal studies suggest that egg-derived antigens induce a sustained and dominant immune response that mediates granuloma formation and liver fibrosis [30,31]. The diagnostic serology of schistosome infection is difficult owing to the multiple antigens that the parasite possesses and excretes/secretes in the circulating blood of infected patients [32]. Thus, the inclusion of egg-derived peptides may provide a higher positivity rate for peptide-based ELISA, which has a high serological specificity and efficiency compared with classical serological techniques. In this study, four peptides from *S. japonicum* were selected, and we explored their potential immunogenicity and diagnostic value, which are essential for understanding the *S. japonicum* molecular biology and an important tool for the diagnosis of schistosomiasis. 

Heat shock proteins (HSPs) are composed of a large number of molecular chaperones, which can be divided into several families, such as HSP27, HSP40, HSP60, HSP70, HSP90, and HSP110, according to their molecular weights and sequence homology [33]. One of their roles is specifically in promoting host–parasite interactions, and they can modulate the adhesion of surface proteins of the parasite to the host cell to facilitate efficient host cell invasion [34]. In schistosomes, HSPs are overexpressed when parasites are exposed to significant levels of stress [35]. The HSPs secreted by *S. japonicum* eggs can induce strong immunomodulatory effects, including immune stimulation and immunosuppression [10]. One important egg protein from *S. japonicum* is the egg-derived heat shock protein 90α (Sjp90α), which has been found in schistosomula, adult worms, and eggs in our previous work [10]. However, the potential immunological role of Sjp90α-derived peptides remains largely unclear. 

The immunodominant antigens of the invasive pathogen are considered prime immune responses, and the highly specific and sensitive antigens were required for the accurate immunodiagnosis of the infection [36]. Some peptides from HSPs are effective inducers of innate and adaptive immunity [37,38]. They activate dendritic cells (DCs) through Toll-like receptors (TLRs) and possess a major role in MHC antigen processing and presentation [39]. In order to better explore the immunogenicity of Sjp90α-derived peptides to the host, we used two software to predict their epitopes. The present study about the identification of epitopes yielded four peptide sequences, and only the Sjp90α-1 peptide could induce increases in DCs and Tfh cells in splenocytes. Then, we confirmed that Sjp90α-1 induced CD4^+^ T-cell differentiation into Tfh cells through BMDCs. Studies showed that the differentiation of CD4^+^ T cells by antigen-presenting cells (APCs) depended on cell–cell contact as well as on soluble factors [40,41,42]. However, whether Sjp90α-1-activated BMDCs drive CD4^+^ T-cell differentiation through the production of cytokines and chemokines or direct cell–cell contact needs to be investigated further. Since pattern recognition receptors (PRRs) are crucial for sensing pathogenic microorganisms and launching innate responses and adaptive immunity during infection [43], which receptor Sjp90α-1 predominantly recognizes and induces in the cellular cascade still needs to be explored. Moreover, the precise *in vivo* effect of the Sjp90α-1 peptide, especially its susceptibility to enzymatic degradation and chemically lability [44], remains to be further elucidated in animal models. Furthermore, modifications for boosting the properties of peptides are still an issue [45], and future studies may provide new information on the Sjp90α-1 peptide related to this important concept. 

It was found that Tfh cells are critical for inducing B-cell differentiation into plasma cells through producing IL-21 and resultantly promote IgG production [46,47]. Whether Sjp90α-1 can significantly induce CD4+ T-cell differentiation into Tfh cells and then promote B-cell differentiation through IL-21 production requires further investigation.

Based on these findings, the Sjp90α-1-derived peptide was chosen to prepare an Sjp90α-1-peptide-based antibody. The result of an Sjp90α-1-peptide-based ELISA showed its potential diagnostic value for schistosomiasis in the early stages of infection. We speculate that the possible reason for this is that Sjp90α is abundantly expressed in the adult tegument and can possibly induce a related antibody response. The tegument forms a protective barrier that shields the parasites from the host immune system and interacts with the host during immune evasion and in nutrient uptake [48,49]. Interestingly, we found that Sjp90α is more abundantly expressed in the tegument of female worms than males using the Sjp90α-1-peptide-based antibody. This is different from the previous study [10], which showed that Sjp90α has low expression in the tegument of male and female worms with Sjp90α-1 antibodies made from recombinant Sjp90α proteins, suggesting the different recognition epitopes of antibodies from peptide and protein. Also, the differences in Sjp90α expression may be related to gender. Studies have shown that the tegument exhibits sex-specific differences in the tegument proteome of adult paired *S. japonicum* [50], and the enhanced expression of certain HSPs in the paired female may play a role in promoting and maintaining sexual maturation in the female worm [51], which needs further investigation. In our study, there is a slight binding at 55+ kDa. Considering heat shock protein 90 has approximately 90 kDa size [52], we speculate the potentials to be identified in two bands, future study is needed to confirm. There are some limitations in our study. The Sjp90α-1-peptide-based polyclonal antibody we prepared may have a weak recognition of unspecific proteins, which needs to be confirmed in the future. Moreover, this peptide-based antibody needs to be effective in human sera in the future.

In conclusion, the Sjp90α-derived peptides of *S. japonicum* were exclusively identified, and the Sjp90α-1 peptide was found to induce CD4^+^ T-cell differentiation through promoting DC activation, which enriched our understanding of the immunopathogenesis of schistosomiasis. Furthermore, the Sjp90α-1 peptide may present a promising immunodiagnostic marker for schistosomiasis.

## 4. Materials and Methods

### 4.1. Mice and Infection

Six-week-old female C57BL/6J mice were purchased from Nanjing Medical University and were maintained in the Animal Laboratory Resource Facility at Nanjing Medical University. Each mouse was infected with 12 ± 1 cercariae of *S. japonicum* (Jiangsu Institute of Parasitic Disease, Wuxi, China) by abdominal skin exposure. All experiments were performed in strict accordance with the Regulations for the Administration of Affairs Concerning Experimental Animals (1988.11.1) and were approved by the Institutional Animal Care and Use Committee (IACUC) of Nanjing Medical University for the use of laboratory animals (IACUC-101025-1). All mice were euthanized via diethyl ether-induced anesthesia for further study.

### 4.2. Immunogenic Peptide Designs from Sjp90α and Conjugation to Carrier

KGKCSVAADNGPTVAPC from the C-terminal part of the Sjp90α protein was designed by the Immune Epitope Database Analysis Resource (IEDB, www.iedb.org, accessed on 15 March 2021) and named Sjp90α-1. We also selected three peptides based on Kolaskar and Tongaonkar (http://imed.med.ucm.es/Tools/antigenic.pl, accessed on 15 May 2022) to predict antigenicity, named Sjp90α-2, Sjp90α-3, and Sjp90α-4. The peptides were synthesized by a commercial company and identified by HPLC-MS/MS. After that, we used an Endotoxin Removal Kit (Yeasen Biotechnology, Shanghai, China) to remove the endotoxin (concentration < 0.1 EU/mL). Sjp90α-1 was the most antigenic peptide and was separately conjugated to Keyhole Limpet Hemocyanin (KLH) and bovine serum albumin (BSA, Sigma-Aldrich, Milwaukee, WI, USA) using m-maleimidobenzoyl-N-hydroxysuccinimide ester (MBS, Thermo Scientific, Rockford, IL, USA). A cysteine residue was added to the C-terminus end of Sjp90α-1 to facilitate conjugation to the carrier protein. An Sjp90α-1-peptide-based antibody was made by Abclonal Biotechnology (Wuhan, China).

### 4.3. Flow Cytometry

Splenocytes were purified from mice, and trypan blue exclusion was used for cell viability assessment (>95% viable cells). Then, the cells were counted and stained for markers following the flow cytometry protocols. 

For the macrophage analysis, 2 × 10^6^ cells of a single-cell suspension were surface-stained with rat antimouse F4/80-PE/Cy7 (eBioscience, San Diego, CA, USA), rat antimouse CD11b-FITC (BD Pharmingen, San Diego, CA, USA), rat antimouse MHC-II-APC (BD Pharmingen), and rat antimouse CD86-BV421 (BD Pharmingen). For the B-cell analysis, 2 × 10^6^ cells of a single-cell suspension were surface-stained with rat antimouse CD19-FITC (BD Pharmingen). For the dendritic cell analysis, 2 × 10^6^ cells of a single-cell suspension were surface-stained with rat antimouse CD11c-Percp-cy5.5 (eBioscience) and rat antimouse MHC-II-APC (BD Pharmingen). For the NK cell analysis, 2 × 10^6^ cells of a single-cell suspension were surface-stained with rat antimouse CD3-Percp-cy5.5 (eBioscience) and rat antimouse NK1.1-BV421(BD Pharmingen). For the Tfh cell analysis, 2 × 10^6^ cells of a single-cell suspension were surface-stained with rat antimouse CD3-Percp-cy5.5 (eBioscience), rat antimouse CD4-FITC (eBioscience), rat antimouse PD1-PE (BD Pharmingen), and rat antimouse CXCR5-PE/Cy7 (BD Pharmingen). 

For the Tregs and Th1/Th2/Th17 analyses, cell preparation and measurements were performed following the published data [53]. All cells were subsequently detected using a FACSVerse flow cytometer (BD Biosciences) and analyzed by FlowJo software (Treestar, Inc., San Carlos, CA, USA).

### 4.4. Collection and Isolation of Serum 

After *S. japonicum* infection, blood samples were collected from each mouse by tail bleeding at 0, 1, 2, 3, 4, and 5 weeks. Once the collection was completed, the blood was immediately centrifuged at 2000 rpm for 20 min after standing at room temperature for half an hour. Then, the supernatant was taken and saved at −80 °C for later use. The serum was collected to evaluate the immune responses to the immunogens without the confounding effects of other antigens.

### 4.5. Enzyme-Linked Immunosorbent Assay (ELISA)

A 96-well flat-bottom microtiter plate (Sigma-Aldrich) was coated with the Sjp90α-1 peptide (10 µg/mL, 100 µL/well), immediately sealed with a plate membrane, and placed in an incubator at 2−8 °C overnight or 37 °C for 2 h. Plates were washed twice with 300 µL of lotion (phosphate-buffered saline (PBS) containing 0.05% Tween-20) and blocked with 1% BSA (Sigma-Aldrich) in each well at 37 °C for 2 h. After another series of washing with 300 µL of lotion. All the diluted serum samples were diluted with PBS at a ratio of 1:10 (100 µL/well), added to the well, and incubated at 37 °C for 2 h. Plates were washed five times with PBS (100 µL/well). Goat antimouse IgG (H+L) (1:10,000, 100 µL/well) (Sigma-Aldrich) was added and incubated at 37 °C for 1 h. After washing, a chromogenic substrate (Multi Sciences, Hangzhou, China) (100 µL/well) was added to each well and incubated for 10 min in the dark. The reaction was stopped by the addition of a stop solution (Multi Sciences) (100 µL/well). The stop solution covered the micropores evenly, which could completely inhibit enzyme activity. Then, readings were taken immediately after adding the stop solution or the samples were placed in the dark at 2–8 °C for 1 h. The optical density (OD) values of the samples at the 450 nm maximum absorption wavelength and a 570 nm or 630 nm reference wavelength were determined to ensure high accuracy. Serum samples from noninfected mice were evaluated to determine the cutoff value from the mean OD values of known negative sera + two standard deviations [54].

### 4.6. Splenocyte Isolation and the Generation of Bone-Marrow-Derived DCs (BMDCs)

Splenocytes were isolated from C57BL/6 mice after putting a 200-mesh nylon net into PBS, crushed with the needle core of a syringe, and centrifuged at 1500 rpm for 5 min. The sediment was treated with a red blood cell lysis solution for 3–5 min and washed twice with PBS. The splenocytes were cultured in Roswell Park Memorial Institute (RPMI) 1640 medium (Gibco, Palo Alto, CA, USA) (containing glutamine) with 10% fetal bovine serum and a 1% penicillin–streptomycin solution (PS). Bone marrow cells were first isolated from the femurs and tibias of C57BL/6 mice. The obtained cells were seeded in 24-well culture plates in the medium. To induce BMDC differentiation, 20 ng/mL granulocyte macrophage colony-stimulating factor (GM-CSF, Pepro Tech, Cranbury, NJ, USA) was added. All cultures were fed by replacing half of the medium and cytokines on days 3 and 5. On day 7, the cells were collected for further use.

### 4.7. BMDC/CD4^+^ T-Cell Cocultures

CD4^+^ T cells were isolated from mouse splenocytes by negative selection using a CD4^+^ T-cell isolation kit (STEMCELL Technologies, Vancouver, Canada) and resuspended in fresh complete medium at a density of 4 × 10^5^ cells/mL. BMDCs were washed twice and cocultured in medium with CD4^+^ T cells in 12-well plates (direct coculture) at a BMDC/CD4^+^ T cell ratio of 1:1 by combining 4 × 10^5^ BMDCs (seeded in the apical inserts). On the second day of coincubation, flow cytometry was used to detect CD4^+^ T-cell differentiation. 

### 4.8. Dot Blot Hybridization (DB)

The antigenic peptide was diluted to 50 ng/µL by PBS, and 2 µL of the corresponding 50 ng/µL peptide diluent was taken from each small square drawn on a nitrocellulose (NC) membrane by a reverse siphon with a 2.5 µL pipe and placed in the center of a 1 cm × 1 cm square cell on the membrane (white), that is, the peptide-covering amount was 100 ng. After finishing the sampling, the NC film was put into an oven at 37 °C for 30 min. Then, the peptide antibody prepared above was closed for 1 h and diluted with the blocking solution at ratios of 1:1000, 1:5000, 1:10,000, 1:50,000, 1:100,000, and 1:200,000. The sealing solution was washed off, and 1 mL of the corresponding primary antibody diluent was added to each well. Then, the plate was incubated for 2 h at room temperature. After washing, 1 mL of an enzyme-conjugated secondary antibody (goat antirabbit HRP, Sigma-Aldrich) diluted at 1:8000 was added to each well and incubated for 1 h. The plates were washed five times then exposed to an ECL solution. The experiment was performed by Abclonal Biotechnology (Wuhan, China). 

### 4.9. Preparation of Soluble Egg Antigen (SEA)

Schistosome soluble egg antigen (SEA) was prepared based on previously described methods [55]. Briefly, New Zealand rabbits were infected with about 1500 *S. japonicum* cercariae by the abdomen. The rabbits were killed 6 weeks after infection. Parasitic eggs were isolated from the liver by enzymatic digestion using 0.05% collagenase B (Sigma-Aldrich). The eggs were suspended in 1 × PBS, frozen and thawed several times, and centrifuged at 4 °C and 15,000× *g* for 30 min. The suspensions (SEA) were collected and sterilized with a 0.22 µm filter. The endotoxin in the suspensions was removed by a ToxOut™ Rapid Endotoxin Removal Kit (Biovision, Milpitas, CA, USA). The residual endotoxin in the SEA extracts was determined using a Pierce LAL Chromogenic Endotoxin Quantitation Kit (Thermo Scientific) according to the manufacturer’s instructions. Finally, the concentration of SEA was tested with a bicinchoninic acid (BCA) Protein Assay Kit (Sigma-Aldrich).

### 4.10. Western Blot 

Antibody verification was performed using Western blot. The laboratory-preserved Sjp90α prokaryotic expression protein was separated by SDS polyacrylamide gel electrophoresis and transferred to a polyvinylidene fluoride (PVDF) membrane (Merck Millipore, MA, USA) at 300 mA for 60 min. The SEA egg antigen was used as a positive control. The membranes were blocked with 5% skim milk in TBST (25 mmol/L Tris base, 150 mmol/L NaCl, and 0.1% Tween 20) for 2 h and incubated overnight at 4 °C with the Sjp90α-1-peptide-based antibody (1:1000). The membranes were washed with TBST and probed with horseradish peroxidase (HRP)-conjugated antirabbit IgG (Sigma-Aldrich) (1:5000) for 1 h at RT. The blots were developed with an enhanced chemiluminescence (ECL) system (Merck Millipore), and images were photographed using a ChemiDocTM Touch Imaging System (Bio-Rad, Hercules, CA, USA). 

### 4.11. Immunofluorescence Assay

*S. japonicum* eggs or adult worms isolated from infected mice were fixed in 4% paraformaldehyde for 30 min, embedded in paraffin, and sectioned at 3 µm. Sections were deparaffinized with xylene for two washes of 15 min each and rehydrated in a 100, 95, 70% ethanol series and deionized water for 5 min each then put in 10 mM sodium citrate buffer (pH 6.0) (Beyotime Biotechnology, Haimen, China), which was brought to a boil and maintained at a sub-boiling temperature for 10 min for antigen retrieval. After cooling, the sections were washed three times with PBS and permeabilized with 0.03% Triton X-100 (Sigma-Aldrich) for 30 min at room temperature. Unspecific binding was blocked with 5% bovine serum albumin (Sigma-Aldrich) for 1 h at room temperature. Sections of eggs and worms were incubated with the Sjp90α-1-peptide-based antibody (1:500) at 4 °C overnight. After washing with 1 × PBST, the parasite tissue sections were further incubated with goat antirabbit IgG (H+L) (Alexa Fluor^®^ 647, Abcam, Cambridge, UK) (1:200) for 1 h at room temperature. Cell nuclei were stained with DAPI Fluoromount (Southern Biotech, Birmingham, AL, USA) and observed under a fluorescence microscope (ZEISS, Imager.A2, Oberkochen, Germany).

### 4.12. Statistical Analysis

Statistical analyses were carried out with GraphPad Prism 7. Data are shown as means ± standard error (SEM). Multiple comparisons were performed by one-way ANOVA with the Bonferroni post hoc test for comparisons between two groups. *p* values < 0.05 were considered significant. Significant differences were as follows: *, *p* < 0.05; **, *p* < 0.01; ***, *p* < 0.001. 

## Data Availability

Not applicable.

## Supplementary Materials

The following supporting information can be downloaded at: https://www.mdpi.com/article/10.3390/pathogens11111238/s1, Figure S1: Different effects of Sjp90α-derived peptides on immune cells.
