# Supplementary material for: Identification and Characterization of Antigenic Properties of Schistosoma japonicum Heat Shock Protein 90α Derived Peptides
Source: Pathogens. 2022 Oct 26;11(11):1238. doi: 10.3390/pathogens11111238 (PMC9696693; doi:10.3390/pathogens11111238)
Supplement: Supplementary file 1 [file pathogens-11-01238-s001.zip › pathogens-1927235-supplementary.pdf]

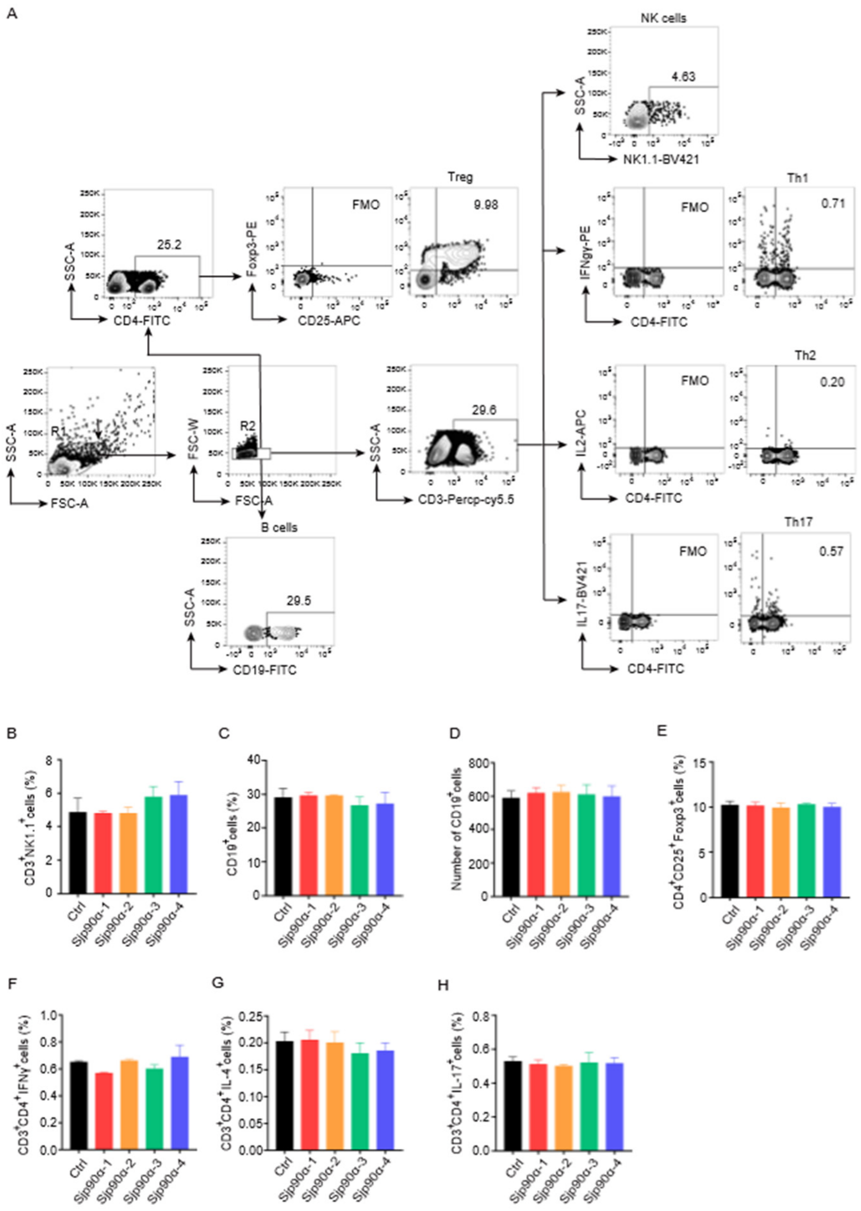

Supplementary Figure S1. Different effects of Sjp90α-derived peptides on immune cells. Mouse splenocytes were treated with Sjp90α-derived peptides (40μg/ml) (n = 4–5 wells, in quadruplication or quintuplicate) or PBS for 24h and analyzed by flow cytometry. The gating strategy (**A**) and statistical chart (**B–H**) showed the frequencies of NK cells ($CD3^+NK1.1^+$), B cells ($CD19^+$), Treg cells ($CD4^+CD25^+Foxp3^+$), and Th1/2/17 cells ($CD3^+CD4^+IFN\gamma^+$/ $CD3^+CD4$+IL-4^+/ $CD3^+CD4^+$IL-17^+). Data were presented as mean ± SEM. **P* < 0.05; ***P* < 0.01; ****P* < 0.001. Data were one representative experiment from two experiments with similar results.
